# Supplementary material for: The Design and Impact of a Clinic-Based Community Program on Food Insecurity, Healthy Eating Behaviors, and Mood
Source: Nutrients. 2023 Oct 10;15(20):4316. doi: 10.3390/nu15204316 (PMC10610369; doi:10.3390/nu15204316)
Supplement: Supplementary file 1 [file nutrients-15-04316-s001.zip › nutrients-2639523-supplementary.pdf]

Design and Impact of a Clinic-based Community Program on Food Insecurity

Tiffany Wesley Ardoin, MD, FACP

Clinic/Community (circle one) **Survey Version:** 1 / 2 / 3 (circle one)

Patients completing this survey have completed consent to participate in the FISH Research Study

Patient Name (last, first) ____________,_____________ DOB _ _ /_ _ / _ _ _ _ MRN# _ _ _ _ _ _ _

Zip Code (circle one) 70802 | 70805 | 70806 | 70807 | 70811 | 70812 | 70815 | Other__________

Initial Screening: USDA Short Form Score:______ CDC Healthy Days Score: 1)_________ 2)___ 3)___ 4)___

When is the last time you saw your PCP? (circle one) Primary Care Provider/Clinic: ________________

Within 1 month / Within 3 months / Within 6 months / Within 12 months / Greater than 1 year or never

Patient Age: 18-24 / 25-34 / 35-44 / 45-54 / 55-64 / 65+ (circle one)

Patient gender: M / F / other (circle one)

Patient race: White / African American or Black / Hispanic / Asian / Other (circle one)

Patient education level: (circle one)

Less than high school

High school or GED

Some College or Vocational School

Completed College

Masters degree or higher

Annual Household Income before taxes: (circle one)

Less than $25,000

$25,000 to $34,999

$35,000 to $49,999

$50,000 to $74,999

$75,000 to $99,999

Greater than $100,000

Number of people in household including yourself: 1 / 2 / 3 / 4 / 5 or more (circle one)

Number of children in household: 1 / 2 / 3 / 4 / 5 or more (circle one)

Do you or anyone in your household receive WIC/EBT? Yes / No (circle one)

Have you used an East Baton Rouge Food Bank? Yes / No (circle one)

Do you have Diabetes? Yes / No (circle one)

Do you have High Blood Pressure? Yes / No (circle one)

Do you have high cholesterol or high lipids? Yes/ No (circle one)

Have you been seen in the ER or admitted to the hospital within the last 6 months? Yes / No (circle one)

If yes, how many times? (circle one) one time / two times / three times / four or more times

Design and Impact of a Clinic-based Community Program on Food Insecurity

Tiffany Wesley Ardoin, MD, FACP

USDA Long Survey

1) Which of these statements best describes the food eaten in your household in the last 12 months: —enough of the kinds of food (I/we) want to eat; —enough, but not always the kinds of food (I/we) want; —sometimes not enough to eat; or, —often not enough to eat?
   - [1] Enough of the kinds of food we want to eat
   - [2] Enough but not always the kinds of food we want
   - [3] Sometimes not enough to eat
   - [4] Often not enough to eat
   - [ ] DK or Refused

2) "(I/We) worried whether (my/our) food would run out before (I/we) got money to buy more." Was that often true, sometimes true, or never true for (you/your household) in the last 12 months?
   - [ ] Often true
   - [ ] Sometimes true
   - [ ] Never true
   - [ ] DK or Refused

3) "The food that (I/we) bought just didn't last, and (I/we) didn't have money to get more." Was that often, sometimes, or never true for (you/your household) in the last 12 months?
   - [ ] Often true
   - [ ] Sometimes true
   - [ ] Never true
   - [ ] DK or Refused

4) "(I/we) couldn't afford to eat balanced meals." Was that often, sometimes, or never true for (you/your household) in the last 12 months?
   - [ ] Often true
   - [ ] Sometimes true
   - [ ] Never true
   - [ ] DK or Refused

5) In the last 12 months, since last (name of current month), did (you/you or other adults in your household) ever cut the size of your meals or skip meals because there wasn't enough money for food?
   - [ ] Yes
   - [ ] No (Skip AD1a)
   - [ ] DK (Skip AD1a)

6) [IF YES ABOVE, ASK] How often did this happen—almost every month, some months but not every month, or in only 1 or 2 months?
   [ ] Almost every month
   [ ] Some months but not every month
   [ ] Only 1 or 2 months
   [ ] DK

7) In the last 12 months, did you ever eat less than you felt you should because there wasn't enough money for food?
   [ ] Yes
   [ ] No
   [ ] DK

8) In the last 12 months, were you every hungry but didn't eat because there wasn't enough money for food?
   [ ] Yes
   [ ] No
   [ ] DK

9) In the last 12 months, did you lose weight because there wasn't enough money for food?
   [ ] Yes
   [ ] No
   [ ] DK

10) In the last 12 months, did (you/you or other adults in your household) ever not eat for a whole day because there wasn't enough money for food?
   [ ] Yes
   [ ] No (Skip AD5a)
   [ ] DK (Skip AD5a)

11) [IF YES ABOVE, ASK] How often did this happen—almost every month, some months but not every month, or in only 1 or 2 months?
   [ ] Almost every month
   [ ] Some months but not every month
   [ ] Only 1 or 2 months
   [ ] DK

Design and Impact of a Clinic-based Community Program on Food Insecurity

Tiffany Wesley Ardoin, MD, FACP

Food Preparation Survey

1. I believe it is important to eat a healthy diet. (circle one)

   I agree / I disagree

2. Do you prepare home cooked meals? (circle one)

   Yes / No

3. Do you prepare meals for people other than yourself regularly? (circle one)

   Yes / No

4. How many times per week do you eat meals outside of your home?

   0-2 / 3-4 / 5-6 / 7 or more

5. How many times per week do you eat home cooked meals?

   0-2 / 3-4 / 5-6 / 7 or more

6. How many servings of fresh fruit do you eat per day?

   0 / 1 / 2 / 3 / 4 / 5 or more

7. How many servings of vegetables do you eat per day?

   0 / 1 / 2 / 3 / 4 / 5 or more

8. How many servings of whole grains do you eat per day?

   0 / 1 / 2 / 3 / 4 / 5 or more

	Not confident at all	Not confident	Confident	Somewhat confident	Very Confident
9. How confident are you in your ability to follow a recipe?					
10. How confident are you in your ability to prepare a healthy meal?					
11. How confident are you in your ability to buy healthy foods at the grocery store?					
12. How confident are you in your knowledge of a healthy diet?					
13. How confident are you in your ability to eat the recommended serving size?					
14. How confident are you in your ability to substitute healthier cooking methods (baking, grilling) for traditionally fried foods?					

In the past month, to what extent have you personally done the following?

	Never do this	Rarely do this	Sometimes do this	Often do this	Always do this
1. Before grocery shopping, I plan my meals that I am shopping for.					
2. I use a list when I go grocery shopping.					
3. When purchasing food, I read the food label and check nutritional values.					

PATIENT HEALTH QUESTIONNAIRE (PHQ-9)

NAME: ______________________ DATE: __________

Over the last *2 weeks*, how often have you been bothered by any of the following problems? *(use "✓" to indicate your answer)*	Not at all	Several days	More than half the days	Nearly every day
1. Little interest or pleasure in doing things	0	1	2	3
2. Feeling down, depressed, or hopeless	0	1	2	3
3. Trouble falling or staying asleep, or sleeping too much	0	1	2	3
4. Feeling tired or having little energy	0	1	2	3
5. Poor appetite or overeating	0	1	2	3
6. Feeling bad about yourself—or that you are a failure or have let yourself or your family down	0	1	2	3
7. Trouble concentrating on things, such as reading the newspaper or watching television	0	1	2	3
8. Moving or speaking so slowly that other people could have noticed. Or the opposite — being so figety or restless that you have been moving around a lot more than usual	0	1	2	3
9. Thoughts that you would be better off dead, or of hurting yourself	0	1	2	3
add columns		+	+	
(Healthcare professional: For interpretation of TOTAL, please refer to accompanying scoring card). TOTAL:				

10. If you checked off *any problems*, how *difficult* have these problems made it for you to do your work, take care of things at home, or get along with other people?	
Not difficult at all	______
Somewhat difficult	______
Very difficult	______
Extremely difficult	______

Copyright © 1999 Pfizer Inc. All rights reserved. Reproduced with permission. PRIME-MD© is a trademark of Pfizer Inc.

A2663B 10-04-2005
